# Welfare on Dairy Cows in Different Housing Systems: Emphasis on Digestive Parasitological Infections

**DOI:** 10.3390/vetsci12020125

**Published:** 2025-02-04

**Authors:** Dragisa Paukovic, Tamara Ilic, Milan Maletic, Nemanja M. Jovanovic, Sreten Nedic, Milorad Mirilovic, Katarina Nenadovic

**Affiliations:** 1 Faculty of Veterinary Medicine, University of Belgrade, Bul. Oslobodjenja 18, 11000 Belgrade, Serbia; paukovicd@sc.rs; 2 Department of Parasitology, Faculty of Veterinary Medicine, University of Belgrade, Bul. Oslobodjenja 18, 11000 Belgrade, Serbia; nmjovanovic@vet.bg.ac.rs; 3 Department of Reproduction, Fertility and Artificial Insemination, Faculty of Veterinary Medicine, University of Belgrade, Bul. Oslobodjenja 18, 11000 Belgrade, Serbia; maletic@vet.bg.ac.rs; 4 Department of Ruminants and Swine Diseases, Faculty of Veterinary Medicine, University of Belgrade, Bul. Oslobodjenja 18, 11000 Belgrade, Serbia; sreten.nedic@vet.bg.ac.rs; 5 Department of Economics and Statistics, Faculty of Veterinary Medicine, University of Belgrade, Bul. Oslobodjenja 18, 11000 Belgrade, Serbia; mija@vet.bg.ac.rs; 6 Department of Animal Hygiene, Faculty of Veterinary Medicine, University of Belgrade, Bul. Oslobodjenja 18, 11000 Belgrade, Serbia; katarinar@vet.bg.ac.rs

**Keywords:** assessment of welfare, cattle, endoparasites, management

## Abstract

The aim of this research was to assess welfare indicators in different dairy cow management systems, determine the prevalence of parasitic infections, and examine the impact of these infections on welfare indicators. This study was conducted in 2024 on 45 Holstein-Friesian cows aged 2 to 6 years (first to third lactation) in Northern Serbia. Monitoring was carried out in tie stall, loose, and pasture-based systems, covering three production phases: late dry period, clinical puerperium, and peak lactation. Cow welfare was evaluated using the Welfare Quality^®^ protocol, and parasitological diagnostics from fecal samples. Identified welfare issues included a low body condition score (BCS), dirtiness of udders, flanks, and legs, integument alterations, nasal and ocular discharge, lameness, and diarrhea. Cows in the pasture-based system had significantly higher scores for dirtiness (*p* < 0.001), while those in tie stalls showed more integument alterations (*p* < 0.001). Loose-housed cows had higher nasal discharge scores (*p* < 0.001). Parasites identified included *Eimeria* spp., *Buxtonella sulcata*, gastrointestinal strongylids, *Moniezia* spp., *Dicrocoelium dendriticum*, *Fasciola hepatica*, and *Paramphistomum* spp. Significant correlations (*p* < 0.001) were found between certain welfare indicators and parasite infections, such as a low BCS with *Eimeria* oocysts and nasal discharge and hairless patches with *Buxtonella sulcata* and *Dicrocoelium dendriticum*. These data indicate needs for improving dairy cows’ welfare and the implementation of effective parasite control measures in all housing systems.

## Simple Summary

The housing system is an important factor affecting the welfare and health of dairy cows. On the other hand, parasites are usually neglected among the etiological agents that affect the poor health status of cattle. However, they can lead to lower productivity and affect animal performance, which results in significant economic losses. This research aimed to assess welfare indicators in different dairy cow management systems, determine the prevalence of parasitic infections and examine the impact of these infections on welfare indicators. Animals in tie stalls showed significantly more integument alterations, while loose-housed and pasture-based cows had higher nasal discharge and dirtiness scores. Parasites identified included *Eimeria* spp., *Buxtonella sulcata*, gastrointestinal strongylids, *Moniezia* spp., *Dicrocoelium dendriticum*, *Fasciola hepatica,* and *Paramphistomum* spp. Significant correlations were found between certain welfare indicators and parasite infections, such as a low body condition score with *Eimeria* oocysts and nasal discharge and hairless patches with *Buxtonella sulcata* and *Dicrocoelium dendriticum*. Results highlight the effect of parasitological infections on the health and welfare of dairy cows.

## 1. Introduction

Over the past 15 years, the selective breeding of dairy cattle has significantly increased milk production in the European Union from 12 to 15 L to 50 L per day. These demands for increased production, combined with high stocking densities in barns, the overexertion of animals to achieve maximum yields, and the implementation of profit-maximizing measures, have significantly impacted the quality of life and lifespan of dairy cows. The housing system is an important factor affecting the welfare, health, and reproduction of dairy cows [1,2]. Compared to tie stall housing systems, pasture-based systems have a considerably lower prevalence of mastitis [3]. On the contrary, controlling individual food intake is difficult in pasture-based systems, which increases the likelihood of metabolic disorders and disrupts reproductive performance [4]. Access to pastures has a positive effect on locomotion, reduces the occurrence of lameness, and enhances productivity and welfare [5] but poses a risk for exposure to gastrointestinal parasites [1].

Parasitic infections are one of the most significant challenges for profitable cattle production. They impact growth, milk production, reproduction, and immune responses to vaccination [6,7]. These infections result in direct losses [8], negatively affect the health and welfare of dairy cattle [9], and cause enormous annual economic losses [10].

From an economic perspective, the most significant cattle helminths in Western Europe are gastrointestinal nematodes and *Fasciola hepatica* [11,12], while, in warmer zones of Northwestern Europe, *Dictyocaulus viviparus* is prominent [13]. In Serbia, infections with gastrointestinal and lung strongylids, trematodes, cestodes, and protozoa are common [14].

To protect profitability and improve animal welfare, the planned use of anthelmintics and acaricides, along with knowledge of the prevalence of parasitoses and resistance profiles to antiparasitic drugs, is necessary. Implementing strategic parasite control measures—which depend on the production environment, management practices, climate, cattle age, parasite epidemiology, and susceptibility to antiparasitics—can ensure a positive return on investment across all cattle production sectors [10].

In recent years, the negative impact of parasites on the welfare of not only cattle [9] but also other ruminants [15] has been described. Ensuring welfare on dairy farms involves (i) providing good physical health for animals, (ii) minimizing unpleasant “affective states” and allowing physiological pleasures, and (iii) enabling animals to live in a manner natural for their species [16]. However, many common farm management practices include high rates of mastitis [17], permit routine painful procedures without pain mitigation [18], and do not provide conditions for the expression of highly motivated behaviors [19]. These practices compromise welfare and cause stress in animals, which consequently reduces herd productivity [20].

Therefore, the aim of this study is to evaluate animal welfare, determine the presence of parasitic infections, and assess their impact on the welfare of dairy cattle in different housing systems and across three production phases (pre-drying, clinical puerperium, and peak lactation).

## 2. Material and Methods

### 2.1. Research Area

**Figure 1:**
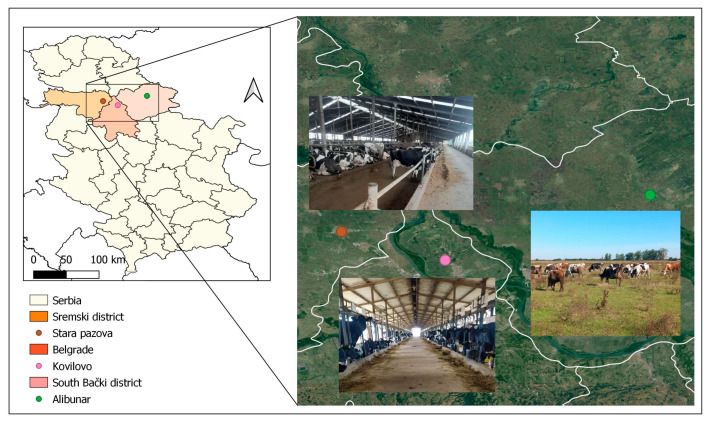
Map of Serbia with administrative districts where the survey is conducted.

This research was conducted from 1 March to 31 August 2024, on dairy farms in the Vojvodina region (45.4° N and 20.2° E). Farms were selected based on accessibility, and traditional cow-rearing practices aligned with the planned study systems: the tie stall system (Kovilovo), loose system (Stara Pazova), and pasture-based system (Alibunar) (Figure 1). This study included 45 Holstein-Friesian cows (15 cows per housing system), aged 2 to 6 years. According to lactation, the examined dairy cows were divided into three age groups: age 2 (cows with 1st lactation), age 3–4 (cows with 2nd lactation), and age 5–6 (cows with 3rd lactation). Assessments of animal welfare and parasitological analyses were performed in three production phases: the late drying-off period (15–20 days before calving), clinical puerperium (28–30 days post-calving), and peak lactation (90 days post-calving). In the tie stall system, cows were kept in the stall with a length between 150 cm and 250 cm and a width between 90 cm and 175 cm. Straw bedding was used (3 kg/head/day or less) and replaced whenever needed. Cows do not have access to pasture, because they have been tied up their entire lives.

The farm with a loose system was closed and had access to a resting area provided with cubicles with conventional rubber mats.

Dairy cows in a pasture-based system had access to pasture from March to November with native grassland nearby the farm and were housed in the stall during the night where straw bedding was used in a small quantity (2 kg/head/day or less).

In all housing systems, automatic cow drinkers were provided. All the drinkers were working correctly. In stalls of the pasture-based system, drinkers were partly dirty (drinkers were dirty, but water was fresh and clean).

Anthelmintic treatment was performed one per year in all farms.

### 2.2. Assessment of Animal Welfare

Cow welfare assessment was carried out at all production phases using the Welfare Quality^®^ Assessment Protocol for Cattle [21]. According to the Welfare Quality^®^ [21], we used resource-based indicators, such as water provision, the cleanliness of water points, water flow, the functioning of water points, the cleanliness of bedding, bedding material, and animal-based indicators, such as the body condition score (BCS), the cleanliness of udders, the cleanliness of flank/upper legs, the cleanliness of lower legs, integument alterations, lameness, discharges, coughs, and diarrhea. Welfare indicators were scored 0 when welfare was good or 2 when welfare was poor and unacceptable. A higher score represented impaired cow welfare. For the body condition assessment, we used the BCS system with a scale from 0 to 2, i.e., 0 if cows have regular body conditions, 1 if cows were very lean, and 2 if cows were very fat. Integumentum alterations scored with 0—absence; and 1—presence of welfare indicators. Lameness scored with 0—not lame; 1—lame; and 2—severely lame animals in the loose and pasture-based housing system, and, in tied animals, 0—not lame; and 2—lame animals [21].

### 2.3. Parasitological Analysis

Fecal samples were collected from each cow from the Ampulla recti. Samples were properly labeled and stored in a portable refrigerator at a temperature of +4 °C. Coprological diagnostics were conducted immediately in a laboratory at the Parasitology Department at the Faculty of Veterinary Medicine, University of Belgrade.

Fecal samples were analyzed using standard parasitological techniques: sodium chloride (NaCl) flotation, sedimentation, the Baermann method, and McMaster method (described in Jovanovic et al. [22]).

For the flotation method, approximately 5 g of sample were thoroughly mixed with a NaCl flotation solution (specific gravity of 1.200). The mixture was then strained through a sieve into a centrifuge tube topped with a cover slip and centrifuged at 1500 RPM for 5 min. Each sample was examined in duplicate. In the sedimentation method, about 5 g of the fecal sample was homogenized in tap water and filtered through a sieve into a conical glass, which was then filled with tap water. After allowing it to stand for 15 min, the supernatant was carefully poured off. This sedimentation process was repeated twice more to clarify the sediment. Three microscope slides were prepared and examined for each sample. For the Baermann method, 30 g of each fecal sample was placed on a sieve with gauze in a funnel and filled with tap water. The sample was left to stand for 24 h. After this period, the first 10 milliliters of fluid was collected into a tube and centrifuged. The supernatant was discarded, and the remaining sediment was examined.

The quantification of the obtained qualitative results was performed using the McMaster method. The method was performed according to the standard procedure for determining the number of parasitic elements in a unit mass (1 g) of feces. With this methodological approach of quantitative FEC, in samples that were positive by qualitative methods, the degree of fecal infection with helminth eggs (EPG—Eggs Per Gramm), oocysts (OPG—Oocysts Per Gramm) and protozoan cysts (CPG—Cysts Per Gramm) was determined.

***Coproculture*:** Fecal samples were pooled and then finely broken using a stirring device and kept moist and brittle; the mixtures were transferred to Petri dishes and placed at 27 °C in an incubator for 7 to 10 days. The culture was kept moist by adding water every 2 days. Finally, larvae were recovered using a modified Baermann technique. The presence of larvae was assessed by using a stereomicroscope, and, when larvae were present, two drops of larval suspension were mixed with one drop of lugol iodine on a glass slide and examined using a stereomicroscope (Carl Zeiss, Jena, Germany) at low magnification power for identification. From each pool submitted to a coproculture, up to 100 third-stage larvae (L3) were morphologically identified according to [23], based on conventional characteristics (total length, esophagus length, tail sheath length, and the number of intestinal cells).

### 2.4. Statistical Analysis

Data were processed with the GraphPad Prism statistic software 7 (GrapfPad Software, San Diego, CA, USA). Results were described by descriptive statistics (mean, standard deviation, and standard error) and as prevalence. The distribution of the welfare indicators was tested by the Kolmogorov–Smirnov distribution fitting test, which showed that distributions were not normal. The differences between welfare indicators were analyzed using the nonparametric Kruskal–Wallis test on the equality of the medians. When significant differences were found, the Dunn–Bonferroni post hoc test was performed. The chi-square (χ^2^) test was used to determine the statistical significance of differences in the welfare indicator and parasitological infection prevalence between cows in different housing systems and the production phase. Relationships between the different welfare indicators and parasitological infections were examined by Spearman’s Rank correlation. For all correlation analyses, the absolute value of Spearman’s correlation coefficients assessed whether weak (|r| < 0.30), moderate (0.30 ≤ |r| < 0.60), or strong (|r| ≥ 0.60) relationships existed, as described by Mason et al. [24]. A 95% confidence interval was established in all tables, with statistical significance set at *p* < 0.05 and *p* < 0.001 levels.

## 3. Results

**Figure 2:**
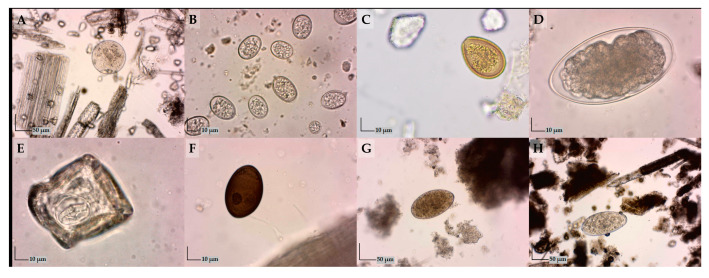
Parasitic elements detected in fecal samples: (**A**) *Buxtonella sulcata* cyst (100×); (**B**) *Eimeria* spp. oocysts (400×); (**C**) *Eimeria bukidnonensis* oocyst (400×); (**D**) Strongylidae egg (400×); (**E**) *Moniezia* spp. egg (400×); (**F**) *Dicrocoelium dendrticum* egg (400×); (**G**) *Fasciola hepatica* egg (100×); and (**H**) *Paramphistomum* spp. egg (100×).

Identified welfare issues in dairy cows were a thin body condition score (BCS), the dirtiness of udders, of flank/upper legs, and of lower legs, integument alternations, nasal discharge, ocular discharge, lameness, and diarrhea. In the examined dairy cows across different housing systems, the protozoa *Eimeria* spp. and *Buxtonella sulcata*, nematodes such as gastrointestinal strongylids, cestodes, from the genus *Moniezia*, and trematodes including *Dicrocoelium dendriticum*, *Fasciola hepatica*, and *Paramphistomum* spp. were diagnosed (Figure 2). Using the coproculture method, three species gastrointestinal strongylids were identified. By isolating and identifying third-stage larvae from samples positive for strongylids, single infections with *Haemonchus* spp. or *Chabertia* spp. (in a pasture-based system) and *Trichostrongylus* spp. (in a loose system) were diagnosed (Supplementary Figure S1).

### 3.1. Analysis from the Aspect of Different Production Phases

#### 3.1.1. Late Drying Phase

A significant difference (*p* < 0.001) was detected in the prevalence of the dirtiness of udders, flank/upper legs, and lower legs between dairy cows in different housing systems, with the higher prevalence in the pasture-based system (46.67%, 7/15; 100%, 15/15) (Table 1). A significant difference (*p* < 0.001) was recorded in the prevalence of integument alternations such as a hairless patch on the neck/shoulder/back between cows, with the higher prevalence in the tie stall system (86.67%, 13/15). A higher prevalence of health problems such as lameness and nasal discharge was noted in the tie stall and loose housing system and were statistically different (*p* < 0.001).

No statistically significant differences were found regarding the mean cow BCS between the different housing systems (Table 2). In dairy cows from the pasture-based system, the mean scores of the dirtiness of udders, flank/upper legs, and lower legs (0.80 ± 0.26, 2.00 ± 0) were significantly higher (*p* < 0.001) than those for cows from tie stall and loose housing systems. In dairy cows from the tie stall housing system, the mean score of integumentum alternations on the neck/shoulder/back (0.93 ± 0.07) were significantly higher (*p* < 0.001, *p* < 0.05) than those for cows from other systems. The mean scores for lameness in cows from the tie stall system (0.67 ± 0.25) were significantly higher (*p* < 0.05) than those for cows from other two systems. In the loose housing system, nasal discharge was significantly higher (*p* < 0.05) than in cows from the pasture system (Table 2).

During this phase, all cows (100%, 15/15) in the tie stall housing system were found to have the ciliate *B. sulcata*. In the loose housing system, *B. sulcata* monoinfection was the most prevalent (80%, 12/15), along with the mixed infections *Eimeria* spp.—*B. sulcata* (40%, 6/15). In the pasture-based system, the most common were *B. sulcata* (86.67%, 13/15) and gastrointestinal strongylids (80%, 12/15), as well as the mixed infections *GIS*—*Eimeria* spp.—*B. sulcata*—*D. dendriticum* (26.67%, 4/15) (Table 3).

A significant difference (*p* < 0.001) was found in the prevalence of gastrointestinal strongylids (80%, 12/15), *Eimeria* spp. (60%, 9/15), *D. dendriticum* (53.33%, 8/15), and the mixed infections *Eimeria* spp.—*B. sulcata* (40%, 6/15) among dairy cows in the late drying phase across different housing systems, with the highest prevalences observed in the pasture-based and loose housing systems. A significant difference (*p* < 0.05) was also noted in the prevalence of the mixed infections *GIS*—*Eimeria* spp.—*B. sulcata*, with the highest prevalence found in cows on pasture (26.67%, 4/15) (Table 3).

Table 4 shows the significant correlations observed between the different cow’s welfare indicators and endoparasites. There was a strong significant correlation between nasal discharge in cows in the loose housing system and *Buxtonella sulcata* (r = −0.61, *p* < 0.05), and between hairless patch in cows in the tie stall system and *Buxtonella sulcata* (r = 0.68, *p* < 0.001).

A significant difference (*p* < 0.05) was detected in the prevalence of a thin BCS between dairy cows in different housing systems, with the higher prevalence in tie stall and loose housing systems (73.33%, 11/15) (Table 5). A significant difference (*p* < 0.001) was detected in the prevalence of the dirtiness of udders, flank/upper legs, and lower legs between dairy cows in different housing systems, with the higher prevalence in the pasture-based system (66.67%, 10/15; 86.67%, 13/15; 86.67%, 13/15) (Table 5). A significant difference (*p* < 0.001, *p* < 0.05) was recorded in the prevalence of integument alternations such as a hairless patch on the neck/shoulder/back, and lameness between cows, with the higher prevalence in the tie stall system (93.33%, 14/15; 40%, 6/15). A significant difference (*p* < 0.05) was detected in the prevalence of lesion/swelling on the tarsus between cows, with the higher prevalence in tie stall and pasture-based systems.

Table 6 shows that the mean scores of thin BCS (0.73 ± 0.12) were significantly higher (*p* < 0.05) from cows in tie stall and loose housing systems than in the pasture-based system (0.33 ± 0.13). In dairy cows on the pasture-based system, the mean scores of dirtiness of udders, flank/upper legs, and lower legs (1.46 ± 0.24, 1.86 ± 0.13, 1.86 ± 0.13) were significantly higher (*p* < 0.001) than those for cows from tie stall and loose housing systems. In dairy cows on the tie stall housing system, the mean scores of integumentum alternations and the hairless patch on neck/shoulder/back (0.93 ± 0.07) were significantly higher (*p* < 0.001, *p* < 0.05) than those for cows from other systems. The mean score of lameness in cows from the tie stall system was significantly higher (*p* < 0.05) compared with cows from other systems (Table 6).

In this phase, cows in the tied housing system were found to have exclusively monoinfections of *B. sulcata* (53.33%, 8/15) and *Eimeria* spp. (46.67%, 7/15), with mixed infections of these protozoa detected in 20% (3/15) of the cows. In the loose housing system, *Buxtonella* was the most prevalent monoinfection (73.33%, 11/15), and the mixed infections *GIS*—*Eimeria* spp.—*B. sulcata* (26.67%, 4/15) were also noted. In the pasture-based system, the most prevalent was the monoinfection with gastrointestinal strongylids (93.33%, 14/15) and the mixed infections *GIS*—*Eimeria* spp.—*B. sulcata*—*D. dendriticum* (26.67%, 4/15) (Table 2).

During the clinical puerperium phase, a significant difference (*p* < 0.001) was found in the prevalence of gastrointestinal strongylids (93.33%, 14/15) and *D. dendriticum* (73.33%, 11/15), with the highest prevalences observed in the pasture-based system. A significant difference (*p* < 0.05) was also noted in the prevalence of the mixed infections *GIS*—*Eimeria* spp.—*B. sulcata*—*D. dendriticum* and *GIS*—*Eimeria* spp.—*D. dendriticum*, which was most common in cows on pasture (26.67%, 4/15) (Table 7).

There was a moderate significant correlation between a thin BCS in cows in the pasture-based system and *Eimeria* spp. (r = −0.53, *p* < 0.05) and between the welfare parameters of a thin BCS in loose system and nasal discharge (r = 0.56, *p* < 0.05) (Table 8). Likewise, a strong significant correlation between a hairless patch in cows in the pasture-based system and *D. dendriticum* (r = 0.66, *p* < 0.001) was observed (Table 8).

#### 3.1.2. Peak of Lactation Phase

A significant difference (*p* < 0.001) was detected in the prevalence of dirtiness on lower legs between dairy cows in different housing systems, with a higher prevalence in loose and pasture-based systems (86.67%, 13/15) (Table 9). A higher prevalence of the hairless patch on the tarsus and lesion/swelling on tarsus was noted in tie stall and loose housing systems and was statistically different (*p* < 0.05).

The mean score of dirtiness on lower legs (1.74 ± 0.18) was significantly higher (*p* < 0.001) than those for cows from the tie stall housing system (Table 10). In dairy cows from the loose system, the mean scores of a hairless patch on the tarsus (0.40 ± 0.13) was significantly higher (*p* < 0.05) than those in the pasture-based system. The mean score of lesion/swelling on the tarsus in cows in the tie stall system (0.53 ± 0.13) was significantly higher (*p* < 0.05) than those in the loose system (Table 10).

In cows in the tie stall housing system, monoinfection with coccidia (40%, 6/15) and mixed infections with *GIS*—*B. sulcata* (26.67%, 4/15) were predominant. In the loose housing system, gastrointestinal strongylids and coccidia (86.67%, 13/15) and the mixed infections *GIS*—*Eimeria* spp.—*B. sulcata* (33.33%, 5/15) were most common. In the pasture-based system, parasitic gastroenteritis was the most prevalent (80%, 12/15), along with the mixed infections *GIS*—*B. sulcata* (20%, 3/15) (Table 3).

Among dairy cows in the peak lactation phase, a significant difference (*p* < 0.001) was found in the prevalence of gastrointestinal strongylids and *Eimeria* spp. (both 86.67%, 13/15), as well as the mixed infections *GIS*—*Eimeria* spp.—*B. sulcata* (33.33%, 5/15), with the highest prevalence observed in the loose housing system. A significant difference (*p* < 0.05) was also noted in the prevalence of *D. dendriticum*, *F. hepatica*, and the mixed infections *GIS*—*Eimeria* spp., with the highest prevalence of dicrocoeliasis in the loose and pasture-based systems (both 40%, 6/15), fascioliasis in the tie stall system (20%, 3/15), and double infections in the loose housing system (20%, 3/15) (Table 11).

Table 12 shows the strong significant correlations observed between a thin BCS and a hairless patch in cows in the pasture-based system (r = 0.66, *p* < 0.001).

Significant differences (*p* < 0.001; *p* < 0.05) were found in the prevalence of *Eimeria* spp. between the cows of different age categories in the dry period and clinical puerperium in tie stall, loose and pasture-based systems with the highest prevalence in cows age 2 (Supplementary Tables S1 and S2). In the pick of lactation, no significant difference was found between the cows of different age categories (Supplementary Tables S1–S3).

Using the McMaster technique, it was found that, in the tie stall system, infection levels with the ciliate *B. sulcata* were low. In the loose system, low levels of infection with coccidia and *B. sulcata* were the most common. In the pasture-based system, diagnoses of infection with gastrointestinal strongylids, coccidia, *D. dendriticum*, and *Paramphistomum* spp. were low levels, while moderate levels of infection with *Moniezia* spp. and *B. sulcata* were found. Only a high level of infection with *B. sulcata* was detected (Supplementary Table S4).

During the clinical puerperium phase, it was observed that, in the tie stall system, protozoan infections involving coccidia and *B. sulcata* remained at low levels. In the loose system, coccidia was detected at low levels, whereas gastrointestinal strongylids ranged from low to moderate. Infection with *B. sulcata* was predominantly high. In the pasture-based system, we noted low levels of coccidia, *B. sulcata*, and *D. dendriticum*, as well as moderate levels of coccidia and gastrointestinal strongylids (Supplementary Table S5).

At the peak lactation phase, the tie stall system showed low-level infections with coccidia, *B. sulcata*, and the trematode *F. hepatica*. In the loose system, low-level infections with coccidia, *B. sulcata*, and gastrointestinal strongylids were identified, while *D. dendriticum* appeared at both low and moderate levels. In the pasture-based system, low-level infections with coccidia, *D. dendriticum*, and *Paramphistomum* spp. were diagnosed, alongside moderate infections with *B. sulcata* and gastrointestinal strongylids (Supplementary Table S6).

## 4. Discussion

In the present study, a thin body condition score (BCS), the dirtiness of udders, of flank/upper legs, and of lower legs, integument alternations, nasal discharge, ocular discharge, lameness, diarrhea, and parasitological infections in all reproductive phases showed high prevalence and should therefore be considered major welfare problems.

The body condition score could be used as one of the significant welfare indicators and a management tool to improve a cow’s nutrition, health, welfare, production, and pregnancy rate because it can provide insight into the current health and welfare status of cows as well as previous management success [25].

In our study, the high prevalence of a thin BCS in dairy cows in all housing systems represents factors affecting welfare in those animals. Many factors have been reported to affect the cow BCS, such as the season of calving [26], stocking rate, level of feeding, diet type [27,28], year season [29], and parasites [13]. We found that a thin BCS in the dry period was more represented in dairy cows in the pasture-based system compared with tie stall and loose systems. According to Washburn et al. [30], the BCS has been shown to be higher in confinement systems than in pasture-based systems. On the other hand, in lactation periods in tied and loose cows, we found a higher prevalence of a thin BCS compared with cows in the pasture-based system. It has been reported that cows lose condition in the beginning of lactation, followed by a slow gain of condition in mid-lactation [31]. High-milking cows in early lactation require a high-energy ration diet to meet their high production needs. This way of feeding very often introduces cows into a state of subacute rumen acidosis (SARA), which can affect the animal’s health and fertility [32]. The data reported in this study indicate inadequate nutrition management in all housing systems.

Parasites are usually neglected among the etiological agents that affect the poor health status of cattle, although they can lead to lower productivity and affect the animal performance, which results in significant economic losses [8]. Our study found a parasitological infection in cattle from all housing systems with a higher prevalence in pasture-based systems. In contrast to the animal welfare benefits of pasture access, numerous epidemiological studies show that grazing is a risk factor for exposure to gastrointestinal parasites [33]. Subclinical gastrointestinal parasitic infections caused lower growth rates, impaired tissue deposition, productivity, and feed intake depress [34]. In the present study, we found that a thin BCS in pasture-based systems correlate moderately with Eimeria spp. According to Daugschies and Najdrowski [35], *Eimeria* spp. causes damage inflicted on the intestinal tissue, so the digestive process and overall homeostasis can become severely affected, negatively affecting animal welfare and performance, even with the absence of clinical disease. We noted that the *Eimeria* spp. were present in all housing systems. These results can be ascribed to the fact that cattle can come into contact with parasites when they feed together in the same areas or when they share food and water [36].

Coccidiosis and buxtonellosis were identified in the examined dairy cows across all rearing systems. Coccidia were diagnosed with prevalences of 35.56% during the late drying phase, 55.56% during the clinical puerperium phase, and 51.11% during the peak lactation phase. In dairy cows during the dry period, a low level of coccidia infection was most common in both loose and pasture-based systems. During the clinical puerperium, low-level infections dominated across all housing systems. At the peak of lactation, coccidia were most prevalent in tie stall housing, although infections remained at a low level. In all housing systems, coccidiosis was more prevalent in younger animals (two years old) than in older animals, where acquired immunity likely offers protection. This immunity mitigates numerous adverse factors, explaining why coccidiosis primarily affects younger cattle yet still occurs sporadically in older animals [37]. The finding of coccidia in 50% of cows aged 5–6 years further supports this observation.

*Buxtonella sulcata* cysts were found in 88.89% of individuals in the late drying phase, 53.33% in the clinical puerperium phase, and 62.22% in the peak lactation phase. These findings significantly differ from previous reports in Serbia, where a lower prevalence of coccidiosis ranging from 6.67% to 24.31% [14,38,39] and buxtonellosis at 15.23% [14] were reported. However, our results align with the prevalence of coccidiosis in cattle (71.24%) in the Mediterranean region [35], where the disease predominantly occurs as mixed infections involving *E. bovis*, *E. ellipsoidalis*, *E. cylindrica*, and *E. zuernii*. These *Eimeria* species cause severe clinical manifestations with negative effects on digestive processes and overall homeostasis [35] and have been confirmed in other countries [40,41].

In our study, *E. bukidnonensis* was also found in cows in the loose housing system, representing the first official record of this coccidium in cattle in Serbia. This finding is of epizootiological importance, as *E. bukidnonensis*, *E. pollita*, and *E. brasiliensis* have not been diagnosed in cattle in the Mediterranean region [41]. The prevalence of buxtonellosis that we determined is notably higher than that reported in cattle in Greece (7.5%) [42] and Bosnia and Herzegovina (27.2%) [43]. However, it is consistent with reports from cattle in [44].

The prevalence of *F. hepatica* in cattle across different regions of Serbia ranged from 5.69% to 12.14% [45] and 15.45% [38]. These rates are slightly higher than our findings, where *F. hepatica* was identified only in cows during the peak lactation phase, with an overall prevalence of 6.67%, and detected only in the tie stall housing system (20%), in the form of low-grade infections. This result, along with the absence of the parasite in the loose and pasture-based systems, can be attributed to variations in climatic and environmental factors and the housing conditions of the examined cows. These factors may include the absence of suitable habitats for the intermediate host or the presence of habitats with insufficiently viable freshwater snails, which do not consistently support the development of infective stages (metacercariae) for the definitive host. Compared to other European countries, our findings are similar to those from Italy [46] but do not align with results from Germany [47] and Ireland [48], which report significantly higher prevalences (27.77–57.10%) of fascioliasis in cattle and dairy cows.

The liver fluke is a common parasite in cattle, affecting milk and meat production, fertility, and overall welfare. Control efforts are complicated by the lack of affordable, accurate on-site diagnostics; challenges related to drug use in lactating cattle; anthelmintic resistance; and climate change, which is expected to significantly alter the parasite’s epidemiology and transmission in the coming decades. The availability of a fully annotated genome map of *F. hepatica* [49] is expected to accelerate progress in these areas. Improved farm-level guidance for parasite control is also essential and will depend on enhanced understanding of the interactions among the environment, ecosystems, snail hosts, wildlife hosts, and farmed animals [50].

*Paramphistomum* spp. were found only in cows in the pasture-based system, with prevalences of 6.67% and 13.33%, and overall prevalences of 2.22% during the late drying phase and 4.44% during the peak lactation phase. These rates are significantly lower compared to previous studies in cattle in Serbia, which reported prevalences of 50.81–92.31% [39,46,51,52]. The prevalence of *Paramphistomum* spp. varies depending on geographic location, ruminant species, sample size, diagnostic procedures, climatic conditions, and rearing systems. In Europe, this trematode infection was reported with a prevalence of 33% in Turkey [53], while, on the Iberian Peninsula, it ranged from 12 to 18.8% [54,55].

*Dicrocoelium dendriticum* was identified with an overall prevalence of 17.78% during the late drying phase and 24.22% during the clinical puerperium phase, exclusively in cows from the pasture-based system (53.33% and 73.33%). During the peak lactation phase, the overall prevalence of this trematode was 33.33%, with 40% of cows from both the loose and pasture-based systems. In the clinical puerperium phase, only *D. dendriticum* was diagnosed among trematodes. This finding partially aligns with previous research in Serbia, which reported this trematode in 20% of cattle [38] and 8.57–14.28% of cows [39]. Dicrocoeliasis has been identified in North Africa, Asia, and Europe, with sporadic cases in North America. Most known clinical cases have been reported in North Africa and the Middle East [56]. This trematode is economically significant, causing substantial annual losses in global livestock production [57]. Due to its zoonotic potential and the possibility that humans—especially children—can become accidentally infected by ingesting infected ants on vegetation or fruits, this trematode also has public health importance [58].

Infections with gastrointestinal strongylids (GIS) were identified in the examined cows, with a prevalence of 26.67% during the late drying phase, 44.44% during the clinical puerperium phase, and 55.56% during the peak lactation phase. These findings are consistent with previous studies in Serbia, which report GIS prevalence ranging from 19.78% to 87.18% [38,39,45,51]. Variations in prevalence may be influenced by factors such as cattle age, location, sampling season, and sample size. Similarly, GIS infections have been reported in cattle worldwide, with a prevalence of 16.6% in intensively farmed dairy cattle and 63.1% in herds in Northern Italy [12]; 14.4% to 25.8% in dairy heifers in Canada [59]; 18.25% in Southern Ethiopia [60]; and 84.24% in Thailand [61]. In dairy cows in the dry period, GIS were diagnosed mostly in the pasture-based system in the form of low- and medium-level infections, in the clinical puerperium phase, moderate-level infections dominated in the loose and pasture-based systems, while, in the peak of the lactation phase, they were most prevalent in the pasture-based system as moderate infections. In the first 18 months of life, cattle develop a strong immunity against GIS. Protection is specific to individual species, so immunity acquired against one type of parasite does not protect the animal against other strongylid species. Immunity primarily ensures that the infection is of lower intensity in older cattle. Along with other factors, it also regulates seasonal fluctuations in the appearance of certain species and their number [37].

Anoplocephalidosis was found only in cows during the late drying phase, with an overall prevalence of 2.22%, exclusively among individuals in the pasture system (6.67%), in the form of moderate infection. This finding aligns with the lower end of prevalence values for this cestodiasis (2.2–78.6%) observed in small and large ruminants in Serbia [38,39,45,51,62]. Reports from Europe indicate that anoplocephalids were detected in 3.4–5.5% of dairy cattle in Poland [63], and 15.5–30.5% of cows in other regions [64]. Researchers emphasize that animal age, the duration of pasture exposure, and the contamination of pastures with infected oribatid mites are crucial factors for the spread and maintenance of cestodes in cattle herds.

The epidemiology of this cestodiasis depends on geographic location, ruminant type, and animal husbandry practices. But it is more common in small ruminants [65]. Differences in prevalence can be attributed to climatic factors, such as the duration of the rainy season, and soil type, as oribatid mites prefer acidic soils. These factors vary by region and influence the dynamics of intermediate host maintenance [66]. Due to the high prevalence of intermediate hosts in wet pasture ecosystems, *Moniezia benedeni* and *M. expansa* remain ongoing concerns for grazing cattle in Europe, Asia, Africa, the Americas, and Australia [67]. In adult dairy cattle, infections are often subclinical, leading to the absence of regular antiparasitic treatments and preventive measures for this cestodiasis within herd health programs [64].

Dirty body parts may reflect animal discomfort that affects the welfare of cows. Significant differences were identified between dairy cows of different housing systems regarding udder, flank, and upper and lower-leg cleanliness, with the highest prevalence of dirtiness in cows in pasture-based systems. Dry cows in pasture-based systems were dirtier than lactating cows in the transition from winter to summer grazing. During the grazing season in the dry periods of cows in the pasture-based system when this study was conducted, the weather had been rainy with wet pasture, which can explain the dirtiness of the cows. These findings align with the results determined by Chincarini et al. [9], who found unclean cows in an organic dairy farm in a period with high precipitation in Central Italy. Also, the risk factor of dirtiness in cows in pasture-based systems could be a farming system, such as the poor hygiene of housing, restriction in space, walking cows from grazing to collecting yards and their exposure to mud and fecal contamination, and straw yards [68]. The body dirtiness of cows is highly related with the occurrence of many health problems such as mastitis [69] and foot disorders [70] and could affect welfare and productivity [71]. To reduce soiling of the udder and other body parts from the manure, cows should not be rushed to and from the milking parlor and feeding area, and the road should be kept clean. The higher dirtiness of cows in lactation compared to the dry periods in tie stalls and loose housing systems might be due to the higher dry matter intakes among lactation cows, resulting in the passage of a greater amount of manure. Early-lactation cows require a high-energy ration diet to meet their high production needs. This way of feeding very often introduces cows into a state of Sub-acute Ruminal Acidosis (SARA), which can lead to significant health problems [32]. Cows with SARA are known to have a much thinner consistency of feces with undigested larger feed particles present. According to Reneau et al. [72], early-lactation cows were dirtier and had greater amounts of manure than late-lactation cows. Since cows are most susceptible to mastitis in early lactation, it is necessary to keep them as clean as possible in this part of the production cycle [72]. The high prevalence of body dirtiness is probably mainly caused due to the failure of daily cleaning routines, regardless of housing system.

According to many authors, integument alterations are a common finding in dairy cattle [70,73]. We found integument alterations of animals in all housing systems with the higher prevalence in cows in tie stall housing systems. Alterations were the most present on the neck/shoulder/back region followed by the hindquarter and tarsus region. Brenninkmeyer et al. [73] reported that alterations such as wounds, swellings, and hairless areas reflect impaired welfare because they are painful and could be a result of interactions between cows and their environment. The causes of occurrence of alterations on the neck region in tie stalls could be due to chains, which constrain the cow’s behavior such as increasing the difficulty of grooming, adopting some lying positions, or reaching the feed [74]. Also, Anderson [75] noted that neck alterations are caused by rubbed or hitting the dorsal aspect of the neck against the underside of the tie-rail while the cow is feeding or rising. Furthermore, our results highlight that the risk of neck/shoulder/back alterations can be reduced when cows are without chains, free to move like in the loose system, and have access to pasture. This result is supported by Alrhmoun et al. [76], who highlighted that the risk of neck, knee, and hock alterations can be reduced when cows have access to pasture. We found strong correlations between hairless patches in tie stalls and pasture cows with *Buxtonella sulcata* and *Dicrocoelium dendriticum*, respectively. We can ascribe these results to the fact that endoparasites negatively affect hosts directly by consuming host resources, causing nutritional deficiencies of proteins, minerals, vitamins and essential fatty acids and damaging host tissues or indirectly by stimulating costly immune responses [77,78]. In our study, we found correlation between a thin BCS and hairless patches in pasture cows, which can be connected with the negative role of endoparasites on animal health. Also, this result is in line with findings reported by Jewell et al. [79], who found an association of a thin BCS with the prevalence of skin alterations in dairy cows.

In many studies, an association between hock lesion and lameness was reported [80,81]. Integument alterations in the tarsus (including hock) were presented in cows in all housing systems, with a higher prevalence (60%, 9/15) in cows in the peak of lactation in tie stall housing systems. Also, the significant difference in lameness was found in tied cows compared with other housing systems. The result of a higher prevalence of tarsus alterations in tied animals could be aligned with a study conducted by Lim et al. [82], who found that cows that had been lame had higher odds of having hock lesions than cows that had not been lame. Additionally, lame cows spend more time resting and have more difficulty standing up or lying down, which increases the time of exposure to the laying surface and risk of collisions with cubicles [83]. Even a number of studies have found a positive effect of pastures on reducing hock lesions [84]; we found a high prevalence of tarsus alterations in pasture cows. This result might be because grazing in this study only has a protective effect, while barn equipment affects the appearance of tarsus alterations. In contrast, the low prevalence of lame animals in pasture systems might be because the pasture provides a more comfortable surface for either lying or standing. In the loose housing system, we found tarsus alterations on cows with a prevalence of 40% (6/15). This result we can ascribe to the fact that the conventional rubber mats in cubicles without bedding, which is used in the examined loose housing system, leads to more hairless areas, crusts, and wounds or swelling in the area of the hocks [80,83]. The data reported in this study indicate uncomfortable resting areas in all housing systems such as inappropriate cubicle dimensions, bedding quality, hardness or abrasive lying surfaces, or colliding with cubicle fittings [80].

According to many authors, nasal and ocular discharge can be indicators of poor health and welfare associated with various respiratory tract diseases [85,86]. In our study, we found nasal discharge in cows in all housing systems with a higher prevalence in the loose housing system (46.67%, 7/15). Also, moderate and strong correlations were found between nasal discharge and a thin BCS and between nasal discharge and *Buxttonela sulcata* in cows in the loose system. These findings may be connected with the fact that many factors may compromise a cow`s immune response such as stress, air quality, parasite infections, and inadequate nutrition [85,87]. In collecting yards in the loose housing system, high levels of ammonia from urine excreted onto manure dirtier floors, noxious gases, and crowded environments could be potential irritants and contributors to infection that lead to nasal discharge [88].

## 5. Conclusions

It is clear from this study that all systems provide negative welfare aspects for dairy cows, with the most impaired cow welfare in tie stall systems. It was shown that the most common causes of further care were a thin BCS, dirtiness, integument alternations (hairless patch, lesions/swelling), lameness, and nasal discharge. In this study, infections by various types of parasites were confirmed in all housing systems. Protozoa, *Eimeria* spp. and *B. sulcata* were predominantly present in the tied-stall system. On the contrary, the highest burden of parasitic infections was confirmed in the pasture-based system. The implementation of strategic parasite control measures is necessary, which could lead to positive economic returns for livestock farmers across all agricultural sectors. Animal welfare should also be an important aspect of dairy farming, and veterinarians need to disseminate knowledge to animal handlers and farmers to minimize the occurrence of impaired welfare and infections. Due to the limited number of animals that were used in experiment, further research with larger samples is recommended.

## Figures and Tables

**Table 1:** Prevalence of the welfare assessment on dairy cows in the late dry period housed in the tie stall, loose, and pasture-based system.

Welfare Parameters		Tie Stall System n = 15		Loose System n = 15		Pasture-Based System n = 15		χ^2^	*p*
		N	% (CI 95%)	N	% (CI 95%)	N	% (CI 95%)		
Thin BCS		7	46.67 (21.41–71.92)	6	40 (15.21–64.79)	8	53.33 (28.08–78.58)	0.54	0.76
Dirtiness	Udders	0	0	0	0	7	46.67 (21.41–71.92)	16.58	0.00 ***
	Flank/upper legs	6	40 (15.21–64.79)	5	33.33 (9.47–57.19)	15	100	16.58	0.00 ***
	Lower legs	0	0	9	60 (35.21–84.79)	15	100	7.5	0.00 ***
Integument alternations	Tarsus (including hock)	4	26.67 (4.29–49.05)	6	40 (15.21–64.79)	2	13.33 (0–30.42)	2.73	0.26
	Carpus	2	13.33 (0–30.42)	0	0	0	0	4.19	0.12
	Neck/shoulder/back	14	93.33 (80.70–100)	2	13.33 (0–30.42)	6	40 (15.21–64.79)	19.62	0.00 ***
	Hindquarter	5	33.33 (9.47–57.19)	6	40 (15.21–64.79)	8	53.33 (28.08–78.58)	1.28	0.53
	Udder	0	**0**	0	0	1	6.67 (0–19.30)	2.05	0.36
Hairless patch	Neck/shoulder/back	13	86.67 (69.46–100)	1	6.67 (0–19.30)	5	20 (0–40.24)	20.41	0.00 ***
	Hindquarter	4	26.67 (4.29–49.05)	4	26.67 (4.29–49.05)	7	46.67 (21.41–71.92)	1.80	0.41
Lesion/swelling	Tarsus (including hock)	1	6.67 (0–19.30)	1	6.67 (0–19.30)	1	6.67 (0–19.30)	/	/
Lameness		5	33.33 (9.47–57.19)	0	0	0	0	11.25	0.00 ***
Nasal discharge		5	33.33 (9.47–57.19)	6	40 (15.21–64.79)	0	0	11.38	0.00 ***
Ocular discharge		3	20 (0–40.24)	1	6.67 (0–19.30)	0	0	3.84	0.15

n—total number of samples; N—number of positive samples; CI–confidence interval; ***—*p* < 0.001

**Table 2:** Mean (±SD) scores of welfare assessment on dairy cows in the late dry period housed in the tie stall, loose, and pasture-based system.

Welfare Parameters		Tie Stall System n = 15	Loose System n = 15	Pasture-Based System n = 15
		Mean (±SD)		
Thin BCS		0.47 ± 0.13	0.40 ± 0.13	0.53 ± 0.13
Dirtiness	Udders	0 ^A^	0 ^B^	0.80 ± 0.26 ^AB^
	Flank/upper legs	0.80 ± 0.26 ^A^	0.67 ± 0.25 ^B^	2.00 ± 0 ^AB^
	Lower legs	0 ^AB^	0.60 ± 0.14 ^A^	2.00 ± 0 ^B^
Integument alternations	Tarsus (including hock)	0.27 ± 0.12	0.40 ± 0.13	0.13 ± 0.09
	Carpus	0.13 ± 0.09	0	0
	Neck/shoulder/back	0.93 ± 0.07 ^A^	0.13 ± 0.09 ^Ab^	0.40 ± 0.13 ^b^
	Hindquarter	0.33 ± 0.13	0.40 ± 0.13	0.53 ± 0.13
	Udder	0	0	0.07 ± 0.07
Hairless patch	Neck/shoulder/back	0.87 ± 0.09 ^Ab^	0.07 ± 0.07 ^A^	0.33 ± 0.13 ^b^
	Hindquarter	0.27 ± 0.12	0.27 ± 0.12	0.47 ± 0.13
Lesion/swelling	Tarsus (including hock)	0.07 ± 0.07	0.07 ± 0.07	0.07 ± 0.07
Lameness		0.67 ± 0.25 ^ab^	0 ^a^	0 ^b^
Nasal discharge		0.67 ± 0.25	0.80 ± 0.26 ^a^	0 ^a^
Ocular discharge		0.40 ± 0.21	0.27 ± 0.17	0

n—total number of samples; ^A, B^—*p* < 0.001; ^a, b^—*p* < 0.05.

**Table 3:** Prevalence of endoparasites on dairy cows in the late dry period housed in the tie stall, loose, and pasture-based system.

Endoparasites	Tie Stall System n = 15		Loose System n = 15		Pasture-Based System n = 15		Total n = 45		χ^2^	*p*
	N	% (CI 95%)	N	% (CI 95%)	N	% (CI 95%)	N	%		
S	0	0	0	0	12	80 (60–100)	12	26.67	32.73	0.000 ***
E	0	0	9	60 (35.21–84.79)	7	46.67 (21.42–71.92)	16	35.56	13.01	0.002 ***
M	0	0	0	0	1	6.67 (0–19.30)	1	2.22	1.03	0.31
B	15	100	12	80 (60–100)	13	86.67 (73.67–99.67)	40	88.89	3.15	0.21
D	0	0	0	0	8	53.33 (28.08–78.58)	8	17.78	19.46	0.000 ***
*p*	0	0	0	0	1	6.67 (0–19.30)	1	2.22	1.03	0.31
Coinfections	N	% (CI 95%)	N	% (CI 95%)	N	% (CI 95%)	N	%	χ^2^	*p*
Double infections										
EB	0	0	6	40 (15.21–64.79)	0	0	6	13.33	13.85	0.000 ***
SB	0	0	0	0	1	6.67 (0–19.30)	1	2.22	2.05	0.36
SD	0	0	0	0	2	13.33 (0–30.53)	2	4.44	4.19	0.12
BP	0	0	0	0	1	6.67 (0–19.30)	1	2.22	2.05	0.36
Triple infections										
SBD	0	0	0	0	2	13.33 (0–30.53)	2	4.44	4.19	0.12
SEB	0	0	0	0	2	13.33 (0–30.53)	2	4.44	4.19	0.12
Quadruple infections										
SEBD	0	0	0	0	4	26.67 (4.29–49.05)	4	8.89	8.78	0.02 *
SEMB	0	0	0	0	1	6.67 (0–19.30)	1	2.22	2.05	0.36

n—total number of samples; N—number of positive samples; CI—confidence interval; ***—*p* < 0.001; *—*p* < 0.05; S—Strongylidae; E—*Eimeria* spp.; B—*Buxtonella sulcata*; M—*Moniezia* spp.; D—*Dicrocoelium dendriticum*; and P—*Paramphistomum* spp.

**Table 4:** Spearman’s Rank correlations between the welfare indicators and endoparasites on dairy cows in the dry period housed in the tie stall and loose system.

Welfare Parameters	Endoparasites	r	*p*
Nasal discharge–Loose system	*Buxtonella sulcata*	−0.61	0.02 *
Hairless patch–Tie stall system	*Buxtonella sulcata*	0.68	0.000 ***

***—*p* < 0.001; *—*p* < 0.05.3.1.2. Clinical Puerperium Phase.

**Table 5:** Prevalence of welfare assessment on dairy cows in clinical puerperium housed in tie stall, loose, and pasture-based system.

Welfare Parameters		Tie Stall System n = 15		Loose System n = 15		Pasture-Based System n = 15		χ^2^	*p*
		N	% (CI 95%)	N	% (CI 95%)	N	% (CI 95%)		
Thin BCS		11	73.33 (50.95–95.71)	11	73.33 (50.95–95.71)	4	26.67 (4.29–49.05)	8.93	0.02 *
Dirtiness	Udders	3	20 (0–40.24)	3	20 (0–40.24)	10	66.67 (42.81–90.53)	9.50	0.000 ***
	Flank/upper legs	6	40 (15.21–64.79)	12	80 (59.76–100)	13	86.67 (69.46–100)	8.92	0.02 *
	Lower legs	4	26.67 (4.29–49.05)	13	86.67	13	86.67 (69.46–100)	16.2	0.000 ***
Integument alternations	Tarsus(including hock)	9	60 (35.21–84.79)	6	40 (15.21–64.79)	8	53.33 (28.08–78.58)	1.25	0.54
	Carpus	2	13.33 (0–30.42)	3	20 (0–40.24)	0	0	3.15	0.21
	Neck/shoulder/back	14	93.33 (80.70–100)	2	13.33 (0–30.42)	6	40 (15.21–64.79)	19.92	0.00 ***
	Hindquarter	8	53.33 (28.08–78.58)	3	20 (0–40.24)	10	66.67 (42.81–90.53)	6.96	0.03 *
	Udder	0	0	0	0	0	0	/	/
Hairless patch	Neck/shoulder/back	14	93.33 (80.70–100)	2	13.33 (0–30.42)	6	40 (15.21–64.79)	19.92	0.00 ***
	Carpus	0	0	3	20 (0–40.24)	0	0	6.42	0.04 *
	Hindquarter	7	46.67 (21.41–71.92)	1	6.67 (0–19.30)	8	53.33 (28.08–78.58)	8.34	0.02 *
lesion/swelling	Tarsus(including hock)	8	53.33 (28.08–78.58)	1	6.67 (0–19.30)	8	53.33 (28.08–78.58)	6.67	0.04 *
Lameness		6	40 (15.21–64.79)	1	6.67 (0–19.30)	1	6.67 (0–19.30)	7.92	0.02 *
Nasal discharge		6	40 (15.21–64.79)	7	46.67 (21.41–71.92)	3	20 (0–40.24)	2.52	0.28
Ocular discharge		2	13.33 (0–30.42)	2	13.33 (0–30.42)	0	0	2.20	0.33
Diarrhea		0	0	2	13.33(0–30.42)	0	0	4.19	0.12

n—total number of samples; N—number of positive samples; CI—confidence interval; ***—*p* < 0.001; *—*p* < 0.05.

**Table 6:** Mean (±SD) scores of welfare assessment on dairy cows in clinical puerperium housed in tie stall, loose, and pasture-based system.

Welfare Parameters		Tie Stall System n = 15	Loose System n = 15	Pasture-Based System n = 15
		Mean (±SD)		
Thin BCS		0.73 ± 0.12 ^a^	0.73 ± 0.11 ^b^	0.33 ± 0.13 ^ab^
Dirtiness	Udders	0.40 ± 0.22 ^A^	0.40 ± 0.22 ^B^	1.46 ± 0.24 ^AB^
	Flank/upper legs	0.80 ± 0.26 ^A^	1.60 ± 0.22	1.86 ± 0.13 ^A^
	Lower legs	0.53 ± 0.24 ^AB^	1.73 ± 0.18 ^A^	1.86 ± 0.13 ^B^
Integument alternations	Tarsus (including hock)	0.60 ± 0.13	0.40 ± 0.13	0.53 ± 0.13
	Carpus	0.13 ± 0.09	0.20 ± 0.11	0
	Neck/shoulder/back	0.93 ± 0.07 ^A^	0.13 ± 0.09 ^Ab^	0.40 ± 0.13 ^b^
	Hindquarter	0.53 ± 0.13	0.20 ± 0.11 ^a^	0.67 ± 0.13 ^a^
	Udder	0	0	0
Hairless patch	Neck/shoulder/back	0.93 ± 0.07 ^A^	0.13 ± 0.09 ^Ab^	0.40 ± 0.13 ^b^
	Carpus	0	0.20 ± 0.11	0
	Hindquarter	0.47 ± 0.13	0.07 ± 0.07 ^a^	0.53 ± 0.13 ^a^
Lesion/swelling	Tarsus (including hock)	0.53 ± 0.13 ^a^	0.07 ± 0.07 ^ab^	0.53 ± 0.13 ^b^
Lameness		0.80 ± 0.26 ^a^	0.07 ± 0.07 ^a^	0.07 ± 0.07 ^a^
Nasal discharge		0.80 ± 0.26	0.47 ± 0.13	0.40 ± 0.22
Ocular discharge		0.27 ± 0.18	0.27 ± 0.18	0
Diarrhea		0	0.27 ± 0.18	0

n—total number of samples; ^A, B^—*p* < 0.001; and ^a, b^—*p* < 0.05.

**Table 7:** Prevalence of endoparasites on dairy cows in clinical puerperium housed in tie stall, loose, and pasture-based system.

**Endoparasites**	**Tie Stall System** **n = 15**		**Loose System** **n = 15**		**Pasture-Based System** **n = 15**		**Total**		**χ^2^**	***p***
	**N**	**%** **(CI 95%)**	**N**	**%** **(CI 95%)**	**N**	**%** **(CI 95%)**	**N**	**%**		
S	0	0	6	40 (15.21–64.79)	14	93.33 (80.70–100)	20	44.44	26.64	0.000 ***
E	7	46.67 (21.42–71.92)	7	46.67 (21.42–71.92)	11	73.33 (50.95–95.71)	25	55.56	2.88	0.24
B	8	53.33 (28.08–78.58)	11	73.33 (50.95–95.71)	9	60 (35.21–84.79)	28	62.22	1.32	0.52
D	0	0	0	0	11	73.33 (50.95–95.71)	11	24.22	29.12	0.000 ***
Coinfections	N	% (CI 95%)	N	% (CI 95%)	N	% (CI 95%)	N	%	χ^2^	*p*
Double infections										
EB	3	20(0–40.24)	1	6.67(0–19.30)	0	0	4	8.89	3.84	0.15
SB	0	0	0	0	2	13.33 (0–30.53)	2	4.44	4.19	0.12
ED	0	0	0	0	1	6.67 (0–19.30)	1	2.22	2.05	0.36
SD	0	0	0	0	1	6.67 (0–19.30)	1	2.22	2.05	0.36
Triple infections										
SEB	0	0	4	26.67 (4.29–49.05)	2	13.33 (0–30.53)	6	13.33	4.61	0.10
SED	0	0	0	0	4	26.67 (4.29–49.05)	4	8.89	8.78	0.02 *
SBD	0	0	0	0	1	6.67 (0–19.30)	1	2.22	2.05	0.36
Quadruple infections										
SEBD	0	0	0	0	4	26.67 (4.29–49.05)	4	8.89	8.78	0.02 *

n—total number of samples; N—number of positive samples; CI—confidence interval; ***—*p* < 0.001; *—*p* < 0.05; S—Strongylidae; E—*Eimeria* spp.; B—*Buxtonella sulcata*; and D—*Dicrocoelium dendriticum.*

**Table 8:** Spearman’s Rank correlations between the welfare indicators and endoparasites on dairy cows in clinical puerperium housed in a pasture-based system.

Welfare Parameters	Endoparasites	r	*p*
Thin BCS–pasture-based system	*Eimeria* spp.	−0.53	0.04 *
Thin BCS–pasture-loose system	Nasal discharge	0.56	0.03 *
Hairless patch–pasture-based system	*Dicrocelium dendriticum*	0.66	0.000 ***

***—*p* < 0.001; *—*p* < 0.05.

**Table 9:** Prevalence of welfare assessment on dairy cows in peak of lactation housed in tie stall, loose, and pasture-based system.

Welfare Parameters		Tie Stall System n = 15		Loose System n = 15		Pasture-Based System n = 15		χ^2^	*p*
		N	% (CI 95%)	N	% (CI 95%)	N	% (CI 95%)		
Thin BCS		10	66.67 (42.81–90.53)	10	66.67 (42.81–90.53)	5	33.33 (9.47–57.19)	4.50	0.11
Dirtiness	Udders	2	13.33 (0–30.42)	5	33.33 (9.47–57.19)	6	40 (15.21–64.79)	2.81	0.24
	Flank/upper legs	7	46.67 (21.41–71.92)	12	80 (59.76–100)	12	80 (59.76–100)	5.18	0.07
	Lower legs	2	13.33 (0–30.42)	13	86.67 (69.46–100)	13	86.67 (69.46–100)	22.88	0.000 ***
Integument alternations	Tarsus (including hock)	9	60 (35.21–84.79)	6	40 (15.21–64.79)	4	26.67 (4.29–49.05)	3.46	0.18
	Carpus	2	13.33 (0–30.42)	0	0	0	0	4.19	0.12
	Neck/shoulder/back	11	73.33 (50.95–95.71)	5	33.33 (9.47–57.19)	7	46.67 (21.41–71.92)	4.98	0.08
	Hindquarter	7	46.67 (21.41–71.92)	5	33.33 (9.47–57.19)	10	66.67 (42.81–90.53)	3.37	0.18
	Udder	0	0	1	6.67 (0–19.30)	0	0	2.05	0.36
Hairless patch	Tarsus(including hock)	4	26.67 (4.29–49.05)	6	40 (15.21–64.79)	0	0	7.20	0.02 *
	Neck/shoulder/back	7	46.67 (21.41–71.92)	5	33.33 (9.47–57.19)	7	46.67 (21.41–71.92)	0.73	0.69
	Hindquarter	6	40 (15.21–64.79)	3	20 (0–40.24)	6	40 (15.21–64.79)	1.80	0.40
Lesion/swelling	Tarsus(including hock)	8	53.33 (28.08–78.58)	2	13.33 (0–30.42)	4	26.67 (4.29–49.05)	5.80	0.05 *
Lameness		5	33.33 (9.47–57.19)	1	6.67 (0–19.30)	2	13.33 (0–30.42)	3.95	0.14
Nasal discharge		3	20 (0–40.24)	2	13.33 (0–30.42)	2	13.33 (0–30.42)	0.34	0.84
Ocular discharge		1	6.67 (0–19.30)	0	0	0	0	2.05	0.36

n—total number of samples; N—number of positive samples; CI—confidence interval; ***—*p* < 0.001; *—*p* < 0.05.

**Table 10:** Mean (±SD) scores of welfare assessment on dairy cows in peak of lactation housed in tie stall, loose, and pasture-based system.

Welfare Parameters		Tie Stall System n = 15	Loose System n = 15	Pasture-Based System n = 15
		Mean (±SD)		
Thin BCS		0.67 ± 0.13	0.67 ± 0.13	0.33 ± 0.13
Dirtiness	Udders	0.27 ± 0.18	0.67 ± 0.25	0.80 ± 0.26
	Flank/upper legs	0.93 ± 0.27	1.60 ± 0.21	1.60 ± 0.21
	Lower legs	0.27 ± 0.18 ^AB^	1.74 ± 0.18 ^A^	1.74 ± 0.18 ^B^
Integument alternations	Tarsus (including hock)	0.60 ± 0.13	0.40 ± 0.13	0.27 ± 0.12
	Carpus	0.13 ± 0.09	0	0
	Neck/shoulder/back	0.73 ± 0.12	0.33 ± 0.13	0.47 ± 0.13
	Hindquarter	0.47 ± 0.13	0.33 ± 0.13	0.67 ± 0.13
	Udder	0	0.07 ± 0.07	0
Hairless patch	Tarsus (including hock)	0.27 ± 0.12	0.40 ± 0.13 ^a^	0 ^a^
	Neck/shoulder/back	0.47 ± 0.13	0.33 ± 0.13	0.47 ± 0.13
	Hindquarter	0.40 ± 0.13	0.20 ± 0.11	0.40 ± 0.13
Lesion/swelling	Tarsus (including hock)	0.53 ± 0.13 ^a^	0.13 ± 0.09 ^a^	0.27 ± 0.12
Lameness		0.33 ± 0.13	0.07 ± 0.07	0.13 ± 0.09
Nasal discharge		0.40 ± 0.22	0.27 ± 0.18	0.27 ± 0.18
Ocular discharge		0.14 ± 0.14	0	0.27 ± 0.18

n—total number of samples; ^A, B^—*p* < 0.001; ^a^—*p* < 0.05.

**Table 11:** Prevalence of endoparasites on dairy cows in peak of lactation housed in tie stall, loose, and pasture-based system.

Endoparasites	Tie Stall System n = 15		Loose System n = 15		Pasture-Based System n = 15		Total		χ^2^	*p*
	N	% (CI 95%)	N	% (CI 95%)	N	% (CI 95%)	N	%		
S	0	0	13	86.67 (73.67–99.67)	11	73.33 (50.95–95.71)	24	53.33	26.25	0.000 ***
E	6	40 (15.21–64.79)	13	86.67 (73.67–99.67)	4	26.67 (4.29–49.05)	23	51.11	11.92	0.003 ***
B	10	66.67 (42.81–90.63)	9	60 (35.21–84.79)	5	33.33 (9.47–57.19)	24	53.33	3.75	0.15
D	0	0	6	40 (15.21–64.79)	6	40 (15.21–64.79)	15	33.33	8.18	0.02 *
F	3	20 (0–40.24)	0	0	0	0	3	6.67	6.43	0.04 *
*p*	0	0	0	0	2	13.33 (0–30.53)	2	4.44	4.19	0.12
Coinfections	N	% (CI 95%)	N	% (CI 95%)	N	% (CI 95%)	N	%	χ2	*p*
Double infections										
SB	4	26.67 (4.29–49.05)	0	0	3	20 (0–40.24)	7	15.56	4.40	0.11
BF	1	6.67 (0–19.30)	0	0	0	0	1	2.22	2.05	0.36
EF	2	13.33 (0–30.53)	0	0	0	0	2	4.44	4.19	0.12
SE	0	0	3	20 (0–40.24)	0	0	3	6.67	6.43	0.04 *
ED	0	0	1	6.67 (0–19.30)	1	6.67 (0–19.30)	2	4.44	1.05	0.59
SD	0	0	1	6.67 (0–19.30)	0	0	1	2.22	2.05	0.36
EB	0	0	1	6.67 (0–19.30)	0	0	1	2.22	2.05	0.36
BD	0	0	0	0	1	6.67(0–19.30)	1	2.22	2.05	0.36
Triple infections										
SEB	0	0	5	33.33 (9.47–57.19)	0	0	5	11.11	11.25	0.000 ***
SBD	0	0	1	6.67 (0–19.30)	0	0	1	2.22	2.05	0.36
SED	0	0	1	6.67 (0–19.30)	2	13.33 (0–30.53)	3	6.67	2.14	0.34
SDP	0	0	0	0	1	6.67 (0–19.30)	1	2.22	2.05	0.36
Quadruple infections										
SEBD	0	0	2	13.33 (0–30.53)	0	0	2	4.44	4.19	0.12
Fivefold infections										
SEBDP	0	0	0	0	1	6.67(0–19.30)	1	2.22	2.05	0.36

n—total number of samples; N—number of positive samples; CI—confidence interval; ***—*p* < 0.001; *—*p* < 0.05; S—Strongylidae; E—*Eimeria* spp.; B—*Buxtonella sulcata*; D—*Dicrocoelium dendriticum*; F—*Fasciola hepatica*; P—*Paramphistomum* spp.

**Table 12:** Spearman’s Rank correlations between the welfare indicators on dairy cows housed in the pasture-based system.

Welfare Parameters		r	*p*
Thin BCS–pasture–based system	Hairless patch	0.66	0.000 ***

***—*p* < 0.001.

## Data Availability

The data presented in this study are available upon request from the corresponding author.

## Supplementary Materials

The following supporting information can be downloaded at www.mdpi.com/article/10.3390/vetsci12020125/s1, Table S1. Prevalence of endoparasites monoinfections on dairy cows in dry period housed in tie stall, loose, and pasture-based system in relation to age; Table S2. Prevalence of endoparasites monoinfections on dairy cows in clinical puerperium housed in tie stall, loose, and pasture-based system in relation to age; Table S3. Prevalence of endoparasites monoinfections on dairy cows in peak of lactation housed in tie stall, loose, and pasture-based system in relation to age; Table S4. Quantitative assessment of fecal samples on dairy cows in dry period housed in tie stall, loose, and pasture-based system; Table S5. Quantitative assessment of fecal samples on dairy cows in clinical puerperium housed in tie stall, loose, and pasture-based system; Table 6. Quantitative assessment of fecal samples on dairy cows in peak of lactation housed in tie stall, loose, and pasture-based system; and Figure S1. The third-stage larvae (L3) recovered using the corpoculture method.
