# Supplementary material for: Welfare on Dairy Cows in Different Housing Systems: Emphasis on Digestive Parasitological Infections
Source: Vet Sci. 2025 Feb 4;12(2):125. doi: 10.3390/vetsci12020125 (PMC11860239; doi:10.3390/vetsci12020125)
Supplement: Supplementary file 1 [file vetsci-12-00125-s001.zip › vetsci-3332931-supplementary.pdf]

Supplementary Table S1. Prevalence of endoparasites monoinfections on dairy cows in dry period housed in tie stall, loose, and pasture-based system in relation to age

Endoparasites	Age 2 years (n=4)		Age 3-4 years (n=6)		Age 5-6 years (n=5)		Total n=15		χ2	p
	**Tie stall system**									
	Age									
	N	%	N	%	N	%	N	%		
B	4	100	6	100	5	100	15	100	8.3	0.14
	**Loose system**									
Endoparasites	Age 2 years (n=4)		Age 3-4 years (n=7)		Age 5-6 years (n=4)		n=15		χ2	p
E	4	100	2	28.57	3	75	9	60	5.93	0.04*
B	4	100	6	85.71	2	50	12	80	3.39	0.18
	**Pasture-based system**									
Endoparasites	Age 2 years (n=5)		Age 3-4 years (n=6)		Age 5-6 years (n=4)		n=15		χ2	p
S	5	100	4	66.67	3	75	12	80	2.44	0.30
E	5	100	0	0	2	50	7	46.67	11.12	0.004***
M	1	20	0	0	0	0	1	6.67	1.61	0.45
B	5	100	4	66.67	4	100	13	86.67	3.46	0.18
D	3	60	4	66.67	1	25	8	53.33	1.81	0.40
P	0	0	1	16.67	0	0	1	6.67	1.61	0.45

*** - p<0.001; * - p<0.05; n - total number of samples; N - number of positive samples; S - Strongylidae; E - *Eimeria* spp.; B - *Buxtonella sulcata*; M - *Moniezia* spp.; D - *Dicrocoelium dendriticum*; P - *Paramphistomum* spp.

Supplementary Table S2. Prevalence of endoparasites monoinfections on dairy cows in clinical puerperium housed in tie stall, loose, and pasture-based system in relation to age

Endoparasites	Age 2 years n=4		Age 3-4 years n=6		Age 5-6 years n=5		Total n=15		χ2	p
	Tie stall system									
	Age									
	N	%	N	%	N	%	N	%		
E	4	100	3	50	0	0	7	46.67	8.97	0.02*
B	1	25	3	50	4	80	8	53.33	2.75	0.25
	Loose system									
Endoparasites	Age 2 years n=4		Age 3-4 years n=7		Age 5-6 years n=4		n=15		χ2	p
S	1	25	2	28.57	3	75	6	40	2.79	0.25
E	1	25	4	57.17	2	50	7	46.67	1.08	0.58
B	3	75	4	57.14	4	100	11	73.33	2.40	0.30
	Pasture-based system									
Endoparasites	Age 2 years n=5		Age 3-4 years n=6		Age 5-6 years n=4		n=15		χ2	p
S	5	100	6	100	3	75	14	93.33	2.95	0.23
E	5	100	4	66.67	2	50	11	73.33	3.07	0.21
B	2	40	5	83.33	2	50	9	60	2.36	0.31
D	4	80	3	50	4	100	11	73.33	3.24	0.20

* - p<0.05; n - total number of samples; N - number of positive samples; S - Strongylidae; E - *Eimeria* spp.; B - *Buxtonella sulcata*; D - *Dicrocoelium dendriticum*;

Supplementary Table S3. Prevalence of endoparasites monoinfections on dairy cows in peak of lactation housed in tie stall, loose, and pasture-based system in relation to age

Endoparasites	Tie stall system									
	Age									
	Age 2 years n=4		Age 3-4 years n=6		Age 5-6 years n=5		Total n=15		χ2	p
	N	%	N	%	N	%	N	%		
E	3	75	2	33.33	1	20	6	40	2.98	0.22
B	2	50	4	66.67	4	80	10	66.67	0.9	0.64
F	2	50	1	16.67	0	0	3	20	3.52	0.17
	Loose system									
Endoparasites	Age 2 years n=4		Age 3-4 years n=7		Age 5-6 years n=4		Total n=15		χ2	p
S	3	75	6	85.71	4	100	13	86.67	1.09	0.58
E	4	100	6	85.71	3	75	13	86.67	1.09	0.58
B	3	75	5	71.43	1	25	9	60	2.79	0.25
D	1	25	3	42.86	2	50	6	40	0.56	0.75
	Pasture-based system									
Endoparasites	Age 2 years n=5		Age 3-4 years n=6		Age 5-6 years n=4		Total n=15		χ2	p
S	3	60	5	83.33	3	75	11	73.33	0.77	0.68
E	1	20	3	50	0	0	4	26.67	3.24	0.20
B	1	20	2	33.33	2	50	5	33.33	0.90	0.64
D	3	60	3	50	0	0	6	40	3.75	0.15
P	1	20	1	16.67	0	0	2	13.33	0.87	0.65

n - total number of samples; N - number of positive samples; S - Strongylidae; E - *Eimeria* spp.; B - *Buxtonella sulcata*; D - *Dicrocoelium dendriticum*; P - *Paramphistomum* spp

Supplementary Table S4. Quantitative assessment of fecal samples on dairy cows in dry period housed in tie stall, loose, and pasture-based system

Housing systems	Degree of infection (Quantitative FEC method)		Endoparasites					
			S	E	M	B	D	P
Tie stall system	**N**		**0**	**0**	**0**	**15**	**0**	**0**
	Low	n	0	0	0	15	0	0
		%	0	0	0	100	0	0
		Mean ±SEM	0	0	0	96.67±15.01	0	0
Loose system	**N**		**0**	**9**	**0**	**12**	**0**	**0**
	Low	n	0	8	0	12	0	0
		%	0	88.89	0	100	0	0
		Mean ±SEM	0	87.50±15.67	0	95.83±15.64	0	0
	Medium	n	0	1	0	0	0	0
		%	0	11.11	0	0	0	0
		Mean ±SEM	0	400	0	0	0	0
Pasture-based system	**N**		**12**	**7**	**1**	**13**	**8**	**1**
	Low	n	10	6	0	2	8	1
		%	83.33	85.71	0	15.38	100	100
		Mean ±SEM	70.00±11.06	108.3±20.07	0	125.0±25.00	93.75±14.75	100
	Medium	n	2	1	1	10	0	0
		%	16.67	14.29	100	76.92	0	0
		Mean ±SEM	250	700	400	495.0±68.50	0	0
	High	n	0	0	0	1	0	0
		%	0	0	0	7.69	0	0
		Mean ±SEM	0	0	0	1050	0	0

Low: <50-200 opg/cpg/epg; Medium: 250-800 opg/cpg/epg; High: >800 opg/cpg/epg (opg/cpg/epg - number of oocysts/cysts/eggs calculated per 1g faeces); N - total number of samples; n - number of positive samples; S - Strongylidae; E - *Eimeria* spp.; M - *Moniezia* spp.; B - *Buxtonella sulcata*; D - *Dicrocoelium dendriticum*; P - *Paramphistomum* spp.

Supplementary Table S5. Quantitative assessment of fecal samples on dairy cows in clinical puerperium housed in tie stall, loose, and pasture-based system

Housing systems	Degree of infection (Quantitative FEC method)		Endoparasites			
			S	E	B	D
Tie stall system	**N**		**0**	**7**	**8**	**0**
	Low	n	0	7	8	0
		%	0	100	100	0
		Mean ±SEM	0	100.0±21.82	93.75±19.90	0
Loose system	**N**		**6**	**7**	**11**	**0**
	Low	n	3	5	1	0
		%	50	71.43	9.09	0
		Mean ±SEM	100.0±50.00	80.00±30.00	100	0
	Medium	n	3	2	7	0
		%	50	28.57	63.63	0
		Mean ±SEM	616.7±88.19	450.0±50.00	550.0±75.59	0
	High	n	0	0	3	0
		%	0	0	27.27	0
		Mean ±SEM	0	0	1033±44.10	0
Pasture-based system	**N**		**14**	**11**	**9**	**11**
	Low	n	5	5	4	11
		%	35.71	45.45	44.44	100
		Mean ±SEM	90.00±18.71	70.00±20.00	162.0±23.94	100.0±16.51
	Medium	n	6	5	3	0
		%	42.86	45.45	33.33	0
		Mean ±SEM	416.7±77.10	490.0±73.14	733.3±44.10	0
	High	n	3	1	2	0
		%	21.43	9.09	22.22	0
		Mean ±SEM	1100±28.87	1100	1100±50.00	0

Low: <50-200 opg/cpg/epg; Medium: 250-800 opg/cpg/epg; High: >800 opg/cpg/epg (opg/cpg/epg - number of oocysts/cysts/eggs calculated per 1g faeces); N - total number of samples; n - number of positive samples; S - Strongylidae; E - *Eimeria* spp.; B - *Buxtonella sulcata*; D - *Dicrocoelium dendriticum*

Supplementary Table S6. Quantitative assessment of fecal samples on dairy cows in peak of lactation housed in tie stall, loose, and pasture-based system

Housing systems	Degree of infection (Quantitative FEC method)		Endoparasites					
			S	E	B	D	F	P
Tie stall system	**N**		**0**	**6**	**9**	**0**	**3**	**0**
	Low	n	0	6	9	0	3	0
		%	0	100	100	0	100	0
		Mean ±SEM	0	100.0±25.82	100.0±18.63	0	83.33±16.67	0
Loose system	**N**		**13**	**13**	**9**	**6**	**0**	**0**
	Low	n	5	7	7	3	0	0
		%	38.46	53.85	77.78	50	0	0
		Mean ±SEM	90.00±29.15	92.86±22.96	128.57±24.05	66.67±16.67	0	0
	Medi um	n	4	6	2	3	0	0
		%	30.77	46.15	22.22	50	0	0
		Mean ±SEM	487.5±96.56	475±85.39	325±75	300±28.87	0	0
	High	n	4	0	0	0	0	0
		%	30.77	0	0	0	0	0
		Mean ±SEM	1012.5±23.94	0	0	0	0	0
Pasture-based system	**N**		**11**	**4**	**5**	**6**	**0**	**2**
	Low	n	3	4	2	6	0	2
		%	27.27		40	100	0	100
		Mean ±SEM	150±28.87	75.00±14.43	175±25	125±28.14	0	75±25
	Medi um	n	7	0	3	0	0	0
		%	63.64	0	60	0	0	0
		Mean ±SEM	464.3±67.89	0	416.7±120.2	0	0	0
	High	n	1	0	0	0	0	0
		%	9.09	0	0	0	0	0
		Mean ±SEM	950	0	0	0	0	0

Low: <50-200 opg/cpg/epg; Medium: 250-800 opg/cpg/epg; High: >800 opg/cpg/epg (opg/cpg/epg - number of oocysts/cysts/eggs calculated per 1g faeces); N - total number of samples; n - number of positive samples; S - Strongylidae; E - *Eimeria* spp.; B - *Buxtonella sulcata*; D - *Dicrocoelium dendriticum*; F - *Fasciola hepatica*; P - *Paramphistomum* spp.

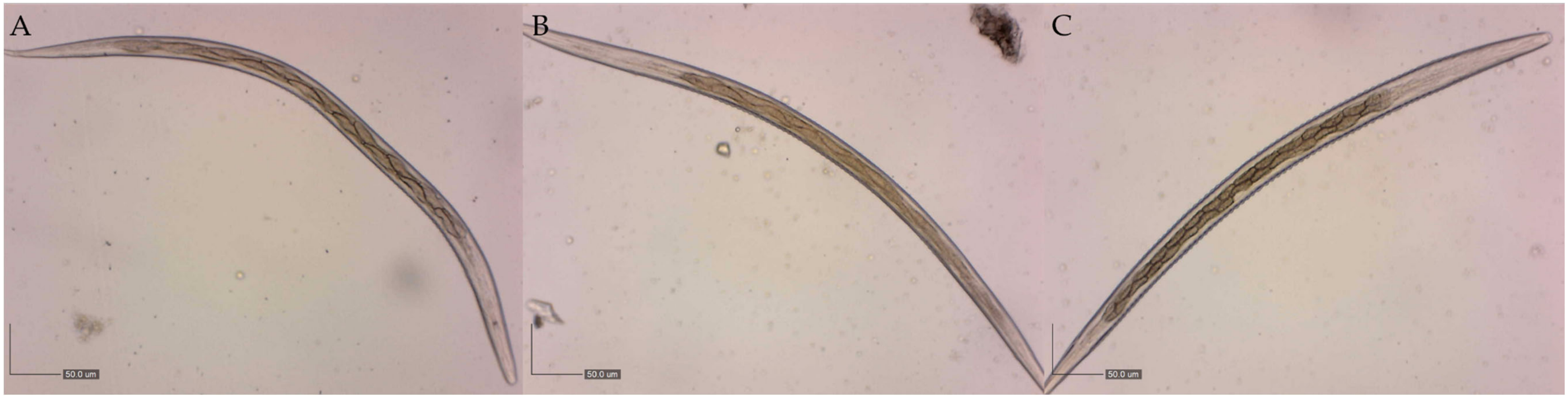

Supplementary Figure S1. The third-stage larvae (L3) recovered using the corpoculture method (40×). (**A**) *Trichostrongylus* spp; (**B**) *Haemonchus* spp; (**C**) *Chabertia* spp. Morphological identification was performed according to total length, esophagus length, tail sheath length, and the number of intestinal cells (Van Wyk and Mayhew 2013).

Van Wyk, J.A. and Mayhew, E., 2013. Morphological identification of parasitic nematode infective larvae of small ruminants and cattle: A practical lab guide. *Onderstepoort Journal of Veterinary Research*, *80*(1), pp.1-14.
